# Supplementary material for: Comparison of Virulence between Two Main Clones (ST11 and ST307) of Klebsiella pneumoniae Isolates from South Korea
Source: Microorganisms. 2022 Sep 13;10(9):1827. doi: 10.3390/microorganisms10091827 (PMC9504348; doi:10.3390/microorganisms10091827)
Supplement: Supplementary file 1 [file microorganisms-10-01827-s001.zip › microorganisms-1907762-supplementary.pdf]

Supplementary Table S1. Primers used in this study

Primers		Sequences (5' to 3' Direction)	T_m (℃)	References
**Detection Primers**	blaKPCtype-F	TGTTGCTGAAGGAGTTGGGC	56	[10]
	blaKPCtype-R	ACGACGGCATAGTCATTTGC	56	
	NDM-F	GGTTTGGCGATCTGGTTTTC	55	[24]
	NDM-R	CGGAATGGCTCATCACGATC	57	
	blaOXA-48-like-F	TGAGCACTTCTTTTGTGATGGCT	61	[10]
	blaOXA-48-like-R	AACGGGCGAACCAAGCATTTT	59	
	CTX-M-F	CRATGTGCAGYACCAGTAA	51	[25]
	CTX-M-R	CGCRATATCRTTGGTGGTG	54	
**Sequencing Primers**	KPC-Full-FW	ATGTCACTGTATCGCCGTCTAGTTCTGCTG	65	This study
	KPC-Full-RV	TTACTGCCCGTTGACGCCCAA	62	
	SEQ-CTX-M-1-FW	AAAAAATCACTGCGCCAGTT	57	This study
	SEQ-CTX-M-1-RV	AAACCGTCGGTGACGATTTT	57	
	SEQ-CTX-M-9-FW	ATGGTGACAAAGAGAGTGCA	55	This study
	SEQ-CTX-M-9-RV	TTACAGCCCTTCGGCGATGA	60	
	SEQ-OXA-48-FW	ATGCGTGTATTAGCCTTATC	51	This study
	SEQ-OXA-48-RV	TTCCTGTTTGAGCACTTCTT	54	
	ref-wzi-RV1	GTGCCGCGAGCGCTTTCTATCTTGGTATTCC	67	[12]
	ref-wzi-RV1	GAGAGCCACTGGTTCCAGAACTTCACCGC	67	
**Virulence gene detection Primers**	rmpA-F	CATAAGAGTATTGGTTGACAG	52	[13]
	rmpA-R	CTTGCATGAGCCATCTTTCA	57	
	p.rmpA-F	TACTTTATATGTAACAAGGATGTAAACATAG	53	[14]
	p.rmpA-R	CAGTAGGCATTGCAGCACTGC	62	
	p.rmpA2-F	CTGTGTCCACTATTGGTGGG	61	[14]

p.rmpA2-R	GATAGTTCACCTCCTCCTCC	60	
kfu-F	GGCCTTTGTCCAGAGCTACG	63	[13]
kfu-R	GGGTCTGGCGCAGAGTATGC	66	
iro-F	GTCCGGCGGTAACTTCAGCC	66	[14]
iro-R	TCAGAATGAAACTACCGCCC	60	
ybtS-F	GACGGAAACAGCACGGTAAA	60	[13]
ybtS-R	GAGCATAATAAGGCGAAAGA	53	
iutA-F	GGGAAAGGCTTCTCTGCCAT	63	[13]
iutA-R	TTATTCGCCACCACGCTCTT	61	
allS-F	CATTACGCACCTTTGTCAGC	58	[14]
allS-R	GAATGTGTCGGCGATCAGCTT	62	
clbA-F	ATGAGGATTGATATATTAATTGGAC	51	[15]
clbA-R	ATTCTGCCCATTTGACGAATG	58	
clbB-F	GATTTGGATACTGGCGATAACCG	60	[15]
clbB-R	CCATTTCCCGTTTGAGCACAC	62	

Supplementary Table S2. Antibiotic susceptibility of the ST11 and ST307 *K. pneumoniae* isolates investigated in this study.

Genotype	Carbapenem	Isolate number	MIC (mg/L)									
			**IMI**	**MRP**	**CPM**	**SXT**	**AZT**	**KAN**	**GEN**	**CIP**	**COL**	**TIG**
ST11	Carbapenem -resistant	SCH2104-31	32	32	>64	>32/604	>64	>64	0.5	>64	0.5	1
		SCH2104-32	32	64	>64	>32/604	>64	2	0.5	>64	0.5	0.5
		SCH2106-16	64	32	>64	0.12/2.4	>64	4	0.5	>64	0.5	0.5
		SCH2107-22	64	64	>64	>32/604	>64	64	64	>64	0.5	0.5
		SCH2108-30	64	32	64	1/18.9	>64	2	0.5	>64	0.5	1
		SCH2104-17	16	16	32	>32/604	>64	16	0.5	>64	0.5	0.5
		SCH2108-43	64	32	>64	>32/604	>64	>64	>64	>64	0.5	0.5
		SCH2108-44	64	16	>64	>32/604	>64	32	0.5	>64	0.5	0.5
	Carbapenem -susceptible	SCH2107-06	1	1	32	2/37.8	>64	>64	>64	>64	64	0.5
		SCH2108-31	0.12	1	32	>32/604	>64	>64	1	>64	1	0.5
		B0706-169	0.25	0.06	16	0.5/9.4	>64	4	0.5	64	0.5	0.5
		B0708-216	0.25	0.06	>64	>32/604	>64	>64	16	>64	0.5	0.5
		K01-Bact-08-03094	0.25	0.06	>64	>32/604	>64	4	0.12	>64	1	0.5
		K01-Bact-08-10058	0.25	0.06	32	>32/604	>64	32	>64	>64	1	0.25
		K01-Bact-08-12164	0.25	0.06	>64	>32/604	>64	4	0.5	>64	1	0.5
		K01-Bact-08-12216	0.25	0.06	>64	>32/604	>64	2	0.5	>64	1	0.5
		K01-Bact-08-12226	0.5	0.06	>64	>32/604	>64	2	0.5	>64	0.5	0.5
		726	0.12	0.06	>64	>32/604	>64	>64	>64	>64	0.5	1
ST307	Carbapenem -resistant	SCH2101-15	32	16	>64	>32/604	>64	64	0.5	>64	0.5	2
		SCH2102-16	32	32	>64	>32/604	>64	32	64	>64	0.5	2
		SCH2102-30	32	32	>64	>32/604	>64	64	>64	>64	0.5	2
		SCH2104-07	64	32	>64	>32/604	>64	64	>64	>64	0.5	2
		SCH2104-33	32	8	>64	>32/604	>64	>64	>64	>64	0.5	2
		SCH2105-10	32	32	>64	>32/604	>64	64	0.5	>64	0.5	2
		SCH2105-20	64	32	>64	>32/604	>64	2	0.5	>64	0.5	4
		SCH2106-08	32	32	>64	>32/604	>64	>64	16	>64	0.5	2
		SCH2108-07	32	32	>64	>32/604	>64	32	>64	>64	0.5	2
		SCH2109-15	32	16	>64	>32/604	>64	64	64	>64	0.5	1
	Carbapenem	SCH2012-07	0.25	0.06	>64	>32/604	>64	32	0.5	>64	0.5	0.5

	-susceptible	SCH2012-19	0.25	0.06	>64	>32/604	>64	32	0.5	>64	0.5	0.5
		SCH2107-01	0.25	0.06	>64	>32/604	>64	32	0.5	>64	0.5	1
		SCH2107-07	1	0.06	>64	>32/604	>64	>64	>64	>64	0.5	1
		SCH2107-08	0.25	0.06	>64	>32/604	>64	32	64	>64	0.5	2
		SCH2107-19	0.25	0.06	0.12	0.25/4.7	0.06	32	1	16	0.5	0.5
		925	2	4	>64	>32/604	>64	64	>64	>64	0.5	2
		SCH-CR31	2	4	>64	>32/604	>64	64	>64	>64	0.5	2
		633	1	2	>64	>32/604	>64	64	>64	>64	0.5	2
		SCH2106-18	0.12	0.06	0.5	>32/604	0.06	32	0.5	>64	0.5	0.5

IMI, imipenem; MRP, meropenem; CPM, cefepime; STX, trimethoprim-sulfamethoxazole; AZT, aztreonam; KAN, kanamycin; GEN, gentamicin; CIP, ciprofloxacin; COL, colistin; TIG, tigecycline.

Resistance was indicated by black background.
